# The Faith, Activity, and Nutrition (FAN) dissemination and implementation study: changes in and maintenance of organizational practices over 24 months in a statewide initiative

**DOI:** 10.1186/s12966-022-01253-9

**Published:** 2022-03-02

**Authors:** Sara Wilcox, Kelsey R Day, Ruth P Saunders, Danielle E Jake-Schoffman, Andrew T Kaczynski, Jessica Stucker, Caroline G Dunn, John A Bernhart

**Affiliations:** 1 Prevention Research Center, Arnold School of Public Health, University of South Carolina, Columbia, SC USA; 2 Department of Exercise Science, Arnold School of Public Health, University of South Carolina, Columbia, SC USA; 3 Department of Health Promotion, Education, and Behavior, Arnold School of Public Health, University of South Carolina, Columbia, SC USA; 4 Department of Health Education and Behavior, College of Health and Human Performance, University of Florida, Gainesville, FL USA

**Keywords:** Physical activity, Nutrition, Faith-based organizations, Ecological model, Intervention

## Abstract

**Background:**

Few studies have examined the impact of ecological health promotion interventions on organizational practices over time, especially in faith-based settings. This statewide dissemination and implementation study examined change in organizational practices and their predictors across a 24-month period, as well as maintenance of change.

**Methods:**

Using a pre-post quasi-experimental design, church coordinators from 92 United Methodist Churches in South Carolina (42% predominantly African American congregations) completed surveys at baseline, and immediate, 12-, and 24-months post-training regarding physical activity (PA) and healthy eating (HE) organizational practices consistent with the Faith, Activity, and Nutrition (FAN) program (opportunities, policies, pastor support, messages) and possible predictors. The study was guided by the RE-AIM framework and the Consolidated Framework for Implementation Research (CFIR). Mixed model repeated measures analyses examined change in organizational practices over time. Regression models examined CFIR predictors of 24-month PA and HE organizational practices, controlling for baseline practices. Churches were also classified as maintainers (implemented at 12 and 24 months), non-sustained implementers (implemented at 12 but not 24 months), delayed implementers (implemented at 24 but not 12 months), and low implementers (implemented at neither 12 nor 24 months) for each FAN component.

**Results:**

PA and HE organizational practices increased over time (*p* < .0001). CFIR domains (and constructs within) of intervention characteristics (adaptability, relative advantage, cost/time), inner setting (relative priority, organizational rewards, readiness, congregant needs), characteristics of the implementer (self-efficacy, perceived benefits), and implementation process (engaging opinion leaders, engaging champions) were important predictors of 24-month PA and HE organizational practices. Over half of churches implementing PA policies, PA messages, HE policies, and HE opportunities at 12 months were maintainers at 24 months, and one-third were maintainers for PA opportunities, HE messages, and PA and HE pastor support. Furthermore, 16% of 12-month non-implementers were delayed implementers at 24 months for PA policies and 31% were delayed implementers for HE policies.

**Conclusions:**

This study makes important contributions to the faith-based health promotion literature by including a large sample of churches, testing an ecological intervention approach, and assessing organizational practices over a 24-month period. Study findings can guide technical assistance and program adaptations over time.

**Trial registration:**

This study was registered in clinicaltrials.gov NCT02868866 on August 16, 2016.

**Supplementary Information:**

The online version contains supplementary material available at 10.1186/s12966-022-01253-9.

## Background

Faith-based organizations are promising settings for public health interventions, as they are widely accessible and represent credible sources of information for their congregants [1–3]. The reach of Christian faith-based organizations is substantial: 65% of American adults describe themselves as Christian, and 62% of Christians report attendance at religious services at least once or twice per month [4]. Furthermore, weekly church attendance is greater among women, older adults, individuals of lower socioeconomic status, and Blacks [4, 5], making churches well-positioned to address health disparities in communities with high rates of chronic disease. 

Although churches are viable settings for health promotion in communities, few studies have examined how to scale up evidence-based programs for broad dissemination and implementation (D&I). Furthermore, little is known about the implementation of church-based interventions. For example, a 2012 review reported that only 28% of church-based health intervention studies measured dose delivered, 27% dose received, 21% both dose delivered and dose received (labeled as implementation), and 9% fidelity [6]. And while program adoption and implementation are critical initial steps for the success of a program, it is also important to examine longer-term implementation and maintenance of programs so that valuable resources are not wasted [7]. The RE-AIM framework defines organizational maintenance as the extent to which a program is sustained over time [8]. Despite its importance, studies reporting organizational program maintenance (sustainability) in behavioral health interventions are rare [9] and are virtually absent in faith-based settings (see exception by [10]). Furthermore, systematic reviews of physical activity (PA) or healthy eating (HE) interventions that apply the RE-AIM framework conclude that reporting of maintenance is low overall [11–17]. 

Interventions in faith-based settings are poised to address policy and systems level changes by engaging church pastors and lay health leaders to make changes to the church environment and practices [1]. However, much of the current literature on faith-based health promotion programs focuses on individual-level outcomes [18–20]. Faith, Activity, and Nutrition (FAN) is a health promotion program that targets policy, systems, and environmental changes (i.e., organizational practices) in churches. FAN is based on the structural model of health behavior [21], and helps churches address four structural components (opportunities, guidelines/policies, pastor support, and messages) to increase PA and HE organization practices. In a large effectiveness trial conducted in South Carolina (SC) churches, FAN was shown to result in large changes to these organizational practices [22], and members reported increases in PA and dietary behavior [23]. As a result, FAN is indexed in the National Cancer Institute’s Evidence-Based Cancer Control Programs [24]. 

In 2014, the potential for sharing the FAN program more widely was tested in a two-phase D&I study, guided by the Reach, Efficacy/Effectiveness, Adoption, Implementation, Maintenance (RE-AIM) framework [8] and the Consolidated Framework for Implementation Research (CFIR) [25]. Organizational practices at both 12 and 24 months were assessed in both phases. The first phase of this study was a group randomized trial conducted in a rural and medically underserved SC county; 42% of county churches adopted the program, and intervention churches reported significantly higher levels of implementation of PA and HE structural components relative to control churches [26, 27]. The results of this trial led to the program being included in the Rural Health Information Hub as a “promising model” [28]. At the 24-month follow-up, the majority of intervention churches were implementing one or more PA and HE component (58% and 97%, respectively) [29]. The second phase of the D&I study was a statewide initiative conducted in partnership with the SC Conference of the United Methodist Church. Adoption and 12-month implementation, as well as factors that predicted both, have been previously reported [30, 31]. In brief, 12% of churches adopted FAN, reach was estimated at 20% of members, and implementation of PA and HE components increased significantly over time.

The focus of the current paper is on organizational practices at 24 months during the statewide initiative of the D&I study. The first aim of this paper was to examine changes over time (baseline, 12 months, 24 months) in PA and HE organizational practices using continuous data. The second aim was to identify factors (guided by CFIR) that influence 24-month organizational practices, controlling for baseline practices. While the CFIR has been widely used in implementation research, Kirk and colleagues argue that its application has lacked depth and that most studies have not investigated CFIR constructs relative to implementation outcomes [32]. The CFIR includes constructs within five domains (intervention characteristics, outer setting, inner setting, individual characteristics, and implementation process) [25] that complement RE-AIM constructs [8]. The third aim was to report maintenance of change in organization practices one-year post-intervention (i.e., at 24 months). Consistent with the RE-AIM framework [33], we defined FAN maintenance as implementation of the FAN organizational practices at both 12 months (immediately post-intervention) and 24 months (one-year post-intervention).

## Methods

### Design

This statewide initiative was a quasi-experimental study. It was reviewed and granted exempt status by the University of South Carolina Institutional Review Board. All 985 churches in the SC Conference of the United Methodist Church were invited to participate via letters, emails, presentations, and other strategies (see more details in [30, 31]). Each interested church identified a coordinator who served as the liaison with the research staff and was responsible for coordinating program implementation (hereafter referred to as FAN Coordinator). A church was eligible for the study if the FAN Coordinator and pastor agreed to participate in evaluation activities. When a pastor served more than one church that wanted to participate, all interested churches were trained, but research staff randomly chose one of the churches to participate in the evaluation. A total of 115 churches were trained in FAN, 93 of which were included in the evaluation. PA and HE organizational practices were assessed at baseline (prior to training) among 92 of the churches, and again at 12 (immediately post-intervention) and 24 months (one-year post-intervention). Constructs from the CFIR model [25] that might influence these organizational practices were assessed at baseline, immediately post-training, 12 months, and 24 months.

### FAN intervention and implementation strategies

The FAN intervention (see also [30]), with its focus on changing church practices, was designed from the onset for organizational maintenance. The intervention focuses on changing four structural components (i.e., organizational practices in the church) based on the structural model of health behavior [21]: providing opportunities, setting guidelines (policies), engaging and supporting pastors, and sharing messages for PA and HE. The primary implementation strategies of FAN, as described in later paragraphs, were training (research staff provided training to the community health advisors who then trained church committees), technical assistance (provided by community health advisors to church committees), tools (identified and/or prepared by research staff and shared with church committees during training), and church committees (implemented the FAN program in their church). The scriptural relevance of physical health from a Christian tradition was incorporated into each strategy.

Training and Implementation Periods. Each participating church formed a committee that attended a one-day training; the committee was responsible for facilitating change in organizational practices consistent with FAN in their churches. During the training, church committees were guided to assess what, if anything, they were doing for each of the four structural components of FAN and to develop a plan to address each. With the exception of several core activities that each church was asked to do (distribute bulletin inserts or handouts, share messages during worship services, distribute educational materials, create a FAN bulletin board, and suggest policies for the church), churches had the flexibility to choose specific activities within each of the structural components that best matched the culture, norms, and preferences of their congregation. During the training, churches were guided to create a 12-month program plan outlining activities they planned to put in place over the implementation period. Intervention materials are available at http://prevention.sph.sc.edu/resources/fan-program-materials.htm. Community health advisors, recruited with assistance from the SC Conference of the United Methodist Church, delivered the trainings and 12 months of technical assistance calls. Details regarding the training they received and implementation fidelity, which was high, have been reported [34]. Up to eight brief calls were delivered to FAN Coordinators (average of 18 min each) and four calls to pastors (average of 17 min each). The semi-structured calls focused on implementation activities for the four structural components, barriers to implementation, and problem solving to overcome barriers. Research staff emailed the FAN Coordinator and pastor monthly program materials as a prompt for use during the first 12 months. All materials were also shared with committees on a USB thumb drive during the training.

#### Maintenance Period

Near the end of the implementation period, community health advisors encouraged the FAN Coordinators and pastors to create a revised program plan (same format as initial 12-month plan) for the upcoming year. Research staff sent 12 monthly emails to pastors and FAN Coordinators in months 13 to 24. These emails included an educational handout, a bulletin insert, and a health-related website to access materials and information about PA and HE.

### Procedure

Baseline, immediate post-training, 12-month, and 24-month surveys were conducted with FAN Coordinators. The church conference also provided basic information about the church such as church size, predominant race of members, and pastor changes. The immediate post-training survey was conducted in person. When possible, the remaining surveys were administered via telephone interviews. Baseline and 12-month interviews were conducted by the Survey Research Laboratory at the University of South Carolina. However, due to the closure of this laboratory, 24-month interviews were conducted by the Center for Public Opinion and Policy Research at Winthrop University. Both groups used a computer-aided telephone interviewing system, and interviewers received specialized training for the study prior to data collection. When FAN Coordinators were not responsive to interview attempts or were unwilling to complete a telephone interview, they were offered the option to complete surveys online or via a mailed paper-and-pencil questionnaire. Interviews and surveys were conducted from February to May of 2017 at baseline (92 by phone, 98.9% response rate); April to July of 2018 at 12 months (80 by phone, 1 online, 3 paper-and-pencil; 90.3% response rate); and April to August of 2019 at 24 months (52 by phone, 16 online, 2 paper-and-pencil; 75.3% response rate).

### Measures

Organizational Practices. FAN Coordinators were asked to report organizational practices in their church, consistent with the four structural components (i.e., organizational practices) of FAN, at baseline (pre-training), 12 months (immediate post-intervention), and 24 months (one-year post-intervention). The four PA organizational practices were assessed with 11 items: 4 for opportunities (2 for integrating PA into existing church events, 1 for offering program(s), and 1 for sharing information about free or low-cost community opportunities), 1 for setting policies, 1 for pastor support (sharing messages during services), and 4 for sharing messages (church bulletins, bulletin boards, others sharing messages during services, and others sharing messages during church meetings and events). The four HE organizational practices were assessed with 9 items: 2 for opportunities (1 for fruits, 1 for vegetables), 2 for setting policies (1 for fruits, 1 for vegetables), 1 for pastor support, and 4 for sharing messages (same categories as for PA). These items were based on the guiding conceptual model [21], used in two prior studies [22, 27], and reviewed by community partners. All items were rated on a 4-point Likert scale for frequency of conducting each activity, with 1 as the least frequent (“rarely or never” or “not at all,” depending on the item) and 4 as the most frequent (“about weekly” or “almost all of the time,” depending on the item). For the policy questions, a score of 3 indicated that the policy was partially in place, whereas a 4 indicated it was fully in place. Mean scores were calculated for multi-item scales. Scores are reported for each structural component and for PA and HE overall composite scores (i.e., mean of the four component scores).

Predictors of Organizational Practices. Additional file 1 presents the CFIR domains, constructs, items, source of each item, as well as when each item was assessed. We included items to measure constructs from the four CFIR domains of (1) intervention characteristics (adaptability, complexity, cost, relative advantage), (2) inner setting (structural characteristics of the church; culture; networks and communication; implementation climate including tension for change, compatibility, relative priority, and organizational incentives/rewards; readiness for implementation; congregant needs), (3) characteristics of the implementer (beliefs; self-efficacy; perceived benefits; individual identification with organization; other self-reported characteristics including duration of church membership, whether they previously led health promotion efforts, age, education, gender, whether they met public health recommendations for PA and fruit and vegetable intake, self-rated health, and body mass index), and (4) implementation process (engaging opinion leaders, engaging champions). Most items, except for those in the other self-reported characteristics area, were rated on a 4-point Likert scale of agreement from strongly disagree (1) to strongly agree (4).

The research team carefully considered when each construct should be assessed. For example, some items could not be assessed at baseline because some knowledge of FAN was needed (e.g., cost, compatibility) or experience with implementation was needed (e.g., congregation’s receptivity to program activities). Some items were assessed at multiple time points because experiences could change over time (e.g., adaptability), whereas other items were expected to be relatively stable over time (e.g., church communication). More details regarding the rationale for using the CFIR, how the CFIR was used to select domains and constructs, and how items were developed is described elsewhere [30].

Finally, we categorized churches according to whether they met criteria, defined a priori, for desirable implementation at 12 and 24 months; a score of 3 or 4 was defined as evidence of desirable implementation. For each of the four structural components, separately for PA and HE, churches were categorized as maintainers if they met criteria at both 12 and 24 months, non-sustained implementers if they met criteria at 12 but not 24 months, delayed implementers if they met the criteria at 24 but not 12 months, and low implementers if they did not meet criteria at either time.

### Data analyses

All analyses were conducted with SAS version 9.4. For Aim 1, we tested change in organizational practices (as continuous variables) over time with repeated measures regression models using mixed linear models (SAS PROC MIXED). These models used all available data (*N* = 92). Separate models were conducted for each structural component and for an overall composite that represented an average of the four structural components, separately for PA and HE. When the time effect was significant, we examined pairwise least square mean differences from baseline to 12 months, baseline to 24 months, and 12 to 24 months. We also computed effect sizes [35] for these comparisons to report magnitude of differences (*d* = 0.20 considered a small effect, 0.50 medium, and 0.80 large).

For Aim 2, we examined the relationships between each CFIR item (or composite index; independent variable) and the 24-month PA or HE composite score for organizational practices (outcome variable) using multiple linear regression models. Each model controlled for corresponding baseline organizational practices. CFIR items were reverse scored, where necessary, so that a higher score always indicated a more favorable rating (e.g., cost). A standardized regression coefficient (*β*), comparable to a correlation coefficient, was computed for each model. Effect sizes of *β* = 0.10 were considered small effects, *β* = 0.30 medium, and *β* = 0.50 large [35]. These models were limited to 70 churches with 24-month data. Given the lack of guidance in the field and literature to draw from, this article focuses on identifying candidate variables for future studies and thus is considered exploratory rather than confirmatory. Multivariate modeling was not deemed helpful given the large number of CFIR constructs, collinearity among them (high variance inflation factor/low tolerance for a sizeable number of variables), and relatively small sample sizes for models given that churches, rather than members, were the unit of study.

In instances where CFIR items were assessed at multiple time points (immediate post-training, 12 months, and/or 24 months), we prioritized the immediate post-training and 12-month assessment, as these time points allowed us to examine the CFIR construct prior to the 24-month assessment, and thus was a predictor of 24-month organizational practices. Nonetheless, because the assessment of CFIR items over time is a unique aspect of the study, we report associations at all time points in a table to allow the reader to understand these relationships.

Finally, for Aim 3, we calculated the percentage of churches classified as maintainers, non-sustained implementers, delayed implementers, and low implementers for each structural component (opportunities, policies, pastor support, and messages), separately for PA and HE. These analyses were limited to the 70 churches with 24-month data.

## Results

### Participating churches

Baseline data were available from the FAN Coordinator in 92 churches. Of these churches, 42% had predominantly African American congregations, 25% had congregations with 500 or more members, 46% reported the presence of a health ministry, and the average tenure of the pastor at baseline was 3.0 (*SD* = 3.4) years. Over the 24-month period, 33% of churches had a change in pastor.

When all baseline (including organizational practices, CFIR ratings, and church characteristics) and immediate post-training variables (CFIR ratings) were compared for FAN Coordinators who completed (*n* = 70) versus did not complete (*n* = 22) the 24-month survey, only one difference was found. FAN Coordinators retained at 24 months were significantly more likely to have been church members for more than three years as compared to those not retained (90.0% vs. 59.1%, *p* < 0.001).

### Changes in PA and HE organizational practices over time

There was a statistically significant time effect for each of the structural components as well as the overall composite scores for PA and HE organizational practices (all *p* values < 0.0001). As shown in Table 1, the mean baseline composite score was 1.45 for PA and 1.85 (out of 4) for HE organizational practices. These scores increased significantly at 12 months (2.11 for PA, 2.62 for HE), and then decreased significantly from 12 to 24 months (1.87 for PA, 2.44 for HE). For each component and composite, the improvement from baseline to 12 months was significant, and all increases were large in magnitude, except for HE opportunities, which was high at baseline and had a significant but moderate increase. For most components, there was a statistically significant reduction from 12 to 24 months, although most of reductions were small or moderate in magnitude. PA and HE policies and HE opportunities did not decrease significantly from 12 to 24 months. For all components and composites, 24-month scores were significantly higher than baseline scores, and effect sizes indicated that churches on average made significant changes to organizational practices from baseline to 24 months that were moderate to large in magnitude.

**Table 1:** Changes in mean physical activity and healthy eating organizational practice scores over time for FAN components (policies, opportunities, pastor support, messages) and composite scores (*N* = 92 churches)

	BL scores			12 M scores		24 M scores		Δ BL to 12 M		Δ BL to 24 M		Δ 12 M to 24 M	
	LSM	SD	SE	LSM	SE	LSM	SE	d	p	d	p	d	p
**Physical activity**													
Composite	1.45	0.47	0.06	2.11	0.06	1.87	0.07	1.40	< .0001	0.88	< .0001	-0.52	< .01
Policies	1.45	0.62	0.11	2.12	0.11	1.92	0.12	1.07	< .0001	0.76	< .01	-0.31	0.18
Opportunities	1.80	0.72	0.07	2.42	0.08	2.23	0.08	0.86	< .0001	0.59	< .0001	-0.27	< .05
Pastor support	1.30	0.66	0.09	1.91	0.09	1.59	0.10	0.92	< .0001	0.43	< .01	-0.48	< .01
Messages	1.26	0.45	0.06	2.00	0.06	1.74	0.07	1.64	< .0001	1.07	< .0001	-0.57	< .001
**Healthy eating**													
Composite	1.85	0.37	0.05	2.62	0.05	2.44	0.06	2.05	< .0001	1.57	< .0001	-0.49	< .01
Policies	1.46	0.66	0.11	2.53	0.11	2.39	0.12	1.60	< .0001	1.39	< .0001	-0.21	0.36
Opportunities	3.40	0.52	0.05	3.73	0.05	3.77	0.06	0.64	< .0001	0.72	< .0001	0.09	0.51
Pastor support	1.26	0.56	0.09	2.01	0.09	1.74	0.10	1.34	< .0001	0.85	< .0001	-0.48	< .05
Messages	1.27	0.46	0.06	2.22	0.06	1.86	0.07	2.05	< .0001	1.26	< .0001	-0.79	< .0001

*Note*: *BL *baseline, *LSM *least square mean, *SD *standard deviation, *SE *standard error, *12 M *12 months, *24 M *24 months, Δ change. Results are from a repeated measures analysis. The composite scores represent a mean of the four FAN component scores. The overall time effect was significant for each model (*p* < .0001) and is not shown in the table (only pairwise p values are shown). Possible scores for each area of implementation can range from 1 to 4, with 4 indicating greater implementation. Cohen's d was calculated as the difference between least square means divided by baseline standard deviation, with *d, * 0.2 considered a small effect, *d, *0.5 a medium effect, and *d, *0.8 a large effect

### Predictors of 24-month organizational practices

Associations between each CFIR item, organized by domain and construct, and 24-month PA and HE organizational practices (composite scores) are shown in Table 2. Within the “intervention characteristics” domain, 12-month ratings of adaptability (can be adapted to fit church) and relative advantage (more effective than other programs) predicted greater PA and HE 24-month practices. Complexity (ease of use and clear/understandable) and cost (time) predicted PA practices. In addition, 24-month ratings of complexity (clear/understandable) and cost (time) were associated with 24-month HE practices. 

Within the “inner setting” domain, 12-month ratings of relative priority (health ministry is as important as spiritual ministry) and readiness for implementation (pastor encouraged congregants to embrace PA components) predicted greater PA and HE practices. Having a predominantly African American congregation, organizational incentives/reward (recognized for implementation), and congregant needs (well-received by most congregants) were predictive of greater PA practices, whereas compatibility (fits with the way you work) and having less than 500 members were predictive of greater HE practices. In addition, 24-month ratings of compatibility were associated with greater PA practices, and organizational incentives/rewards and congregant needs were associated with higher HE practices. The constructs of church culture as well as networks and communication were not related to PA or HE practices.

**Table 2:** Scores for each item, by CFIR domain and construct, and associations with 24-month composite scores (*N* = 70 churches)

		Physical Activity		Healthy Eating	
**CFIR DOMAIN, Construct**, and Item	Time	Mean (SD) or %	^a^Model β	Mean (SD) or %	^a^Model β
**INTERVENTION CHARACTERISTICS**					
**Adaptability**					
-Can be adapted to fit church	PT	3.09 (0.42)	0.08	3.10 (0.43)	-0.02
	12M	3.24 (0.50)	0.21*	3.40 (0.58)	0.29*
	24M	2.96 (0.47)	0.32**	3.31 (0.67)	0.23*
**Complexity**					
-Easy to use	PT	3.13 (0.38)	0.08	3.14 (0.49)	-0.11
	12M	3.16 (0.48)	0.26*	3.39 (0.58)	0.21
	24M	3.01 (0.40)	0.37***	3.35 (0.57)	0.10
-Clear and understandable	PT	3.32 (0.47)	0.12	3.40 (0.49)	-0.09
	12M	3.30 (0.46)	0.23*	3.52 (0.50)	0.16
	24M	3.19 (0.43)	0.28**	3.42 (0.50)	0.23*
**Cost**					
-Expensive (reverse scored)	PT	2.92 (0.59)	0.04	2.85 (0.66)	0.04
	12M	3.04 (0.48)	-0.02	2.60 (0.80)	0.07
	24M	2.98 (0.50)	-0.02	2.82 (0.60)	0.08
-Great deal of time (reverse scored)	PT	2.76 (0.61)	0.04	2.69 (0.61)	0.16
	12M	2.69 (0.66)	0.23*	2.85 (0.58)	0.09
	24M	2.74 (0.59)	0.01	2.70 (0.65)	0.25*
**Relative advantage**					
-More effective than other programs	12M	3.04 (0.56)	0.31**	3.04 (0.56)	0.40**
	24M	2.79 (0.66)	0.36**	2.79 (0.66)	0.24*
**INNER SETTING**					
**Structural characteristics**					
-Presence of health ministry	BL	47.14	0.06	47.14	0.05
-500+ members	C	25.71	-0.18	25.71	-0.28*
-Predominantly African American	C	42.86	0.22*	42.86	0.21
-Pastor change in past year	C	34.29	-0.14	34.29	-0.12
-Tenure of pastor, years	BL	3.00 (3.40)	-0.08	3.00 (3.40)	0.00
^b^**Culture**	BL	3.45 (0.48)	0.13	3.45 (0.48)	0.00
^c^**Networks & Communications**					
-Composite	BL	3.26 (0.40)	0.16	3.26 (0.40)	0.09
-Little tension/conflict	BL	2.89 (0.55)	0.01	2.89 (0.55)	0.02
**Implementation Climate**					
**Tension for change**					
-New ideas readily accepted	BL	2.71 (0.60)	-0.01	2.71 (0.60)	0.03
-Leaders like traditional ways (reverse scored)	BL	2.20 (0.67)	0.05	2.20 (0.67)	0.10
**Compatibility**					
-Matches church priorities	PT	3.10 (0.61)	0.05	3.10 (0.61)	0.04
	12M	2.94 (0.65)	0.18	2.94 (0.65)	0.21
	24M	2.70 (0.61)	0.30**	2.70 (0.61)	0.18
-Fits with way you work	PT	3.35 (0.48)	0.00	3.35 (0.48)	-0.15
	12M	3.14 (0.50)	0.19	3.14 (0.50)	0.27*
	24M	2.92 (0.47)	0.20	2.92 (0.47)	0.13
**Relative priority**					
-Health ministry as important as spiritual ministry	12M	3.04 (0.73)	0.29**	3.04 (0.73)	0.31**
	24M	3.01 (0.81)	0.23*	3.01 (0.81)	0.23*
**Organizational incentives/rewards**					
-Recognized for implementation	12M	3.03 (0.58)	0.29**	3.17 (0.60)	0.17
	24M	2.82 (0.67)	0.32**	2.90 (0.69)	0.30**
**Readiness for implementation**					
-Received enough training	12M	3.13 (0.46)	0.16	3.24 (0.58)	0.18
	24M	3.01 (0.56)	0.32**	3.13 (0.60)	0.05
-Pastor encouraged congregants to embrace	12M	3.15 (0.58)	0.28**	3.18 (0.67)	0.31**
	24M	2.79 (0.69)	0.33**	2.91 (0.77)	0.49***
**Congregant needs**					
-Well received by most congregants	12M	2.80 (0.72)	0.37***	2.97 (0.68)	0.22
	24M	2.69 (0.68)	0.43***	2.92 (0.68)	0.25*
**CHARACTERISTICS OF THE INDIVIDUAL (FAN Coordinator)**					
**Beliefs about the intervention**					
-Valuable for church	PT	3.42 (0.50)	0.07	3.51 (0.50)	0.00
	12M	3.48 (0.50)	0.22*	3.48 (0.50)	0.18
	24M	3.20 (0.56)	0.34**	3.37 (0.52)	0.19
**Self-efficacy**					
-Have skills to make changes	PT	3.09 (0.38)	-0.01	3.17 (0.41)	-0.29*
	12M	3.10 (0.61)	0.37***	3.23 (0.52)	0.19
	24M	2.97 (0.52)	0.32**	3.13 (0.54)	0.25*
-Confident can make (continue to make) changes	PT	3.06 (0.46)	0.21*	3.05 (0.51)	-0.14
	12M	3.00 (0.55)	0.42***	3.11 (0.64)	0.30**
	24M	2.79 (0.56)	0.34***	2.97 (0.68)	0.36**
**Perceived benefits**					
-Church will benefit (has benefited) from changes	PT	3.43 (0.50)	0.00	3.61 (0.49)	-0.19
	12M	3.00 (0.63)	0.33**	3.15 (0.59)	0.23*
	24M	2.87 (0.60)	0.47***	2.98 (0.62)	0.45***
-Worthwhile for me if church makes (continues to make) changes	PT	3.42 (0.50)	-0.02	3.59 (0.52)	-0.09
	12M	3.45 (0.50)	0.20	3.51 (0.53)	0.09
	24M	3.37 (0.54)	0.17	3.38 (0.55)	0.22
**Individual identification with organization**					
-Want to perform to best of ability	BL	3.64 (0.48)	0.10	3.64 (0.48)	0.00
-Feel strong sense of commitment	BL	3.74 (0.44)	0.10	3.74 (0.44)	-0.02
**Other personal attributes**					
-Church membership >3 years	BL	90.00	0.17	90.00	0.01
-Led health promotion efforts	BL	55.71	0.07	55.71	-0.09
-Age, years	BL	57.40 (12.44)	0.00	57.40 (12.44)	0.08
-Some college	BL	90.00	-0.12	90.00	-0.11
-Women	BL	94.29	0.07	94.29	0.11
-Meets public health recommendations for target behavior (PA, HE)	PT	62.32	0.01	28.57	-0.08
	12M	62.12	0.13	44.78	0.21
	24M	57.35	0.01	33.33	0.12
-Self-rated health (5=excellent)	PT	3.66 (0.93)	0.04	3.66 (0.93)	0.02
	12M	3.72 (0.87)	0.18	3.72 (0.87)	0.19
	24M	3.68 (0.83)	0.05	3.68 (0.83)	0.02
-Body mass index, kg/m^2^	PT	28.04 (5.62)	0.13	28.04 (5.62)	0.24*
	12M	28.20 (6.04)	0.13	28.20 (6.04)	0.25*
	24M	28.38 (6.09)	0.12	28.38 (6.09)	0.26*
**IMPLEMENTATION PROCESS**					
**Engaging opinion leaders**					
-Leaders actively involved	12M	2.75 (0.68)	0.37***	2.89 (0.79)	0.38***
	24M	2.69 (0.70)	0.40***	2.76 (0.67)	0.46***
**Engaging champions**					
-At least one person is champion	12M	3.24 (0.55)	0.27*	3.32 (0.58)	0.17
	24M	3.18 (0.64)	0.39***	3.16 (0.68)	0.28*

^*^p < .05. ^**^p < .01. ^***^p < .001

*Note*: For time, *C *reported from church conference prior to starting study, *BL *baseline (pre-training), *PT *immediate post-training, *12 M *12 months, *24 M *24 months

^a^β represents the standardized regression coefficient for each CFIR item predicting 24-month PA and HE maintenance, adjusted for baseline practices

^b^Culture score was an average of 2 items: pastor has a sense of personal responsibility for improving congregant health, pastor is open to changes in practices that impact congregants. Coefficient alpha = 0.74

^c^Composite score for networks and communication was an average of 3 items: pastor and church leaders share information and knowledge, church leaders involve members in decision making, and pastor has good working relationships with other church leaders. Coefficient alpha = 0.69

Within the “characteristics of the individual (FAN Coordinator)” domain, 12-month ratings of self-efficacy and perceived benefits (church has benefited) were predictive of greater PA and HE practices. Beliefs (valuable for church) were predictive of PA practices, whereas having a FAN Coordinator with a higher body mass index was predictive of HE practices. The construct of individual identification with the organization was not related to PA or HE practices.

Within the “implementation process” domain, 12-month ratings of engaging opinion leaders (leaders actively involved) were predictive of PA and HE practices. Engaging champions (at least one person is champion) was predictive of PA practices. Finally, 24-month ratings of engaging champions were associated with HE practices.

### Categorization of 24-month maintenance of PA and HE Components

As shown in Table 3 for PA, 55% of churches that met criteria for desirable implementation of policies at 12 months sustained implementation at 24 months. Furthermore, 16% of churches not implementing policies and 16% not implementing opportunities at 12 months implemented these components at 24 months (delayed implementers). For opportunities and pastor support, 44% and 39% maintained implementation from 12 to 24 months (maintainers). Although the absolute number of churches was small, 56% of churches implementing messages at 12 months continued to implement at 24 months (maintainers). When examined as percentage of the total sample (*n* = 70), the maintenance of policies component was the highest (26%), followed by opportunities (17%) and pastor support and messages (10 and 7%).

**Table 3:** Physical activity maintainers, non-sustained implementers, delayed implementers, and low implementers, based on 12- and 24-month assessments of organizational practices (*N* = 70 churches)

**Churches Implementing at 12 Months**								
	Implementers		Maintainers (implemented at 12 & 24 months)			Non-Sustained Implementers (implemented at 12 but not 24 months)		
	n	% of total sample	n	% of implementers	% of total sample	n	% of implementers	% of total sample
Policies	33	47	18	55	26	15	45	24
Opportunities	27	39	12	44	17	15	56	21
Pastor support	18	26	7	39	10	11	61	20
Messages	9	13	5	56	7	4	44	6
**Churches Not Implementing at 12 Months**								
	Non-Implementers		Delayed Implementers (implemented at 24 but not 12 months)			Low Implementers (low implementers at 12 & 24 months)		
	*n*	% of total sample	*n*	% of non-implementers	% of total sample	*n*	% of non-implementers	% of total sample
Policies	37	53	6	16	9	31	84	41
Opportunities	43	61	7	16	10	36	84	51
Pastor support	52	74	3	6	4	49	94	66
Messages	61	87	0	0	0	61	100	87

*Note*: Churches were categorized according to whether they met a-priori criteria for implementation at 12 and 24 months. A score of 3 (“about monthly” or “some of the time,” depending on the item) or 4 (“about weekly” or “almost all of the time,” depending on the item) was defined as acceptable implementation. These data were limited to the 70 churches where FAN Coordinators completed the 24-month survey

As shown in Table 4 for HE, 97% of churches that met criteria for desirable implementation of opportunities at 12 months continued to implement at 24 months; however, 60 of the 64 (94%) churches met the criteria for desirable implementation at baseline. All four churches not implementing opportunities at 12 months were implementing at 24 months (delayed implementers). For HE policies, 65% that were implementing at 12 months maintained at 24 months, and 31% of those not implementing at 12 months were by 24 (delayed implementers). For pastor support and messages 41% and 33% maintained implementation from 12 to 24 months (maintainers). Of churches not implementing HE components at 12 months, 69–94% were not implementing at 24 months (low implementers). When examined as a percentage of the total sample (*n* = 70), 91% of churches maintained opportunities, 31% maintained policies, 13% maintained pastor support, and 10% maintained messages.

**Table 4:** Healthy eating maintainers, non-sustained implementers, delayed implementers, and low implementers, based on at 12- and 24-month assessments of organizational practices (*N* = 70 churches)

**Churches Implementing at 12 Months**								
	Implementers		Maintainers (implemented at 12 & 24 months)			Non-Sustained Implementers (implemented at 12 but not 24 months)		
	n	% of total sample	n	% of implementers	% of total sample	n	% of implementers	% of total sample
Policies	34	49	22	65	31	12	35	17
Opportunities	66	94	64*	97	91	2	3	3
Pastor support	22	31	9	41	13	13	59	19
Messages	21	30	7	33	10	14	67	20
**Churches Not Implementing at 12 Months**								
	Non-Implementers		Delayed Implementers (implemented at 24 but not 12 months)			Low Implementers (low implementers at 12 & 24 months)		
	n	% of total sample	n	% of non-implementers	% of total sample	n	% of non-implementers	% of total sample
Policies	36	51	11	31	16	25	69	36
Opportunities	4	6	4	100	6	0	0	0
Pastor support	48	69	4	8	6	44	92	63
Messages	49	70	3	6	4	46	94	66

*Note*: Churches were categorized according to whether they met a-priori criteria for implementation at 12 and 24 months. A score of 3 (“about monthly” or “some of the time,” depending on the item) or 4 (“about weekly” or “almost all of the time,” depending on the item) was defined as acceptable implementation. These data were limited to the 70 churches where FAN Coordinators completed the 24-month survey

^a^60 churches met criteria for implementation at baseline

## Discussion

It is critical to scale up health promotion interventions for greater public health impact [36], and while adoption and implementation of these interventions are important initial steps, longer term implementation and maintenance are important for making a population impact [7]. This paper contributes to the literature by examining, within the context of a D&I study, change in organizational practices to promote PA and HE over two-years, predictors of organizational practices, and maintenance of these practices from immediately post-intervention (12 months) to one-year post-intervention (24 months). The paper fills several gaps in the translational and implementation science literatures including the lack of ecological interventions in this setting [18–20], the dearth of health behavior interventions that address organizational maintenance [9, 11–17], and the lack of studies that use constructs from the CFIR to predict implementation outcomes over time [32]. By using both RE-AIM and the CFIR to guide our project, we were able to not only consider factors important for assessing the public health impact of the program (RE-AIM), but also organizational and implementer characteristics (CFIR) that impact longer-term organizational practices. Their combination provides a richer understanding of our program that aims to change organizational practices.

The first aim of this paper was to examine 24-month change in organizational practices using continuous measures. Changes from baseline to 24 months were statistically significant and moderate to large in magnitude for all PA and HE components of FAN, indicating that churches made and sustained meaningful changes after training.

For Aim 3, we calculated the percentage of churches classified as maintainers, non-sustained implementers, delayed implementers, and low implementers for each structural component (opportunities, policies, pastor support, and messages) based on a priori criteria for desirable implementation at 12 and 24 months for PA and HE practices. Maintenance was defined as implementation at both 12 and 24 months and ranged from 33 to 65% for all components except HE opportunities. It was much higher (97%) for HE opportunities, but 94% of churches were implementing at baseline. Furthermore, we documented small but meaningful amounts of delayed implementation for PA policies, PA opportunities, and HE policies. This delayed implementation (i.e., at 24 but not 12 months) may reflect the challenges of installing changes in organizational practices, particularly on relatively short timelines. 

It is difficult to compare our levels of maintenance with other studies because studies of maintenance in faith-based settings are rare—see an exception by [10]—and because there is wide variation in conceptualizing, defining, and measuring maintenance/sustainability [37, 38]. Reviews in community and organizational settings indicate that partial maintenance is common and, when assessed, less than half of projects are continued with high levels of fidelity [37]. Results in school settings are similar [39]. In a faith-based study, the sustainability across churches of 21 possible health activities ranged from 0–67%, with fewer than 40% of churches demonstrating 24-month sustainability for most (16 or 76%) of these activities [10]. 

The PA changes in organizational practices of FAN were maintained at lower levels compared to the HE components, but these results largely reflect lower levels of implementation of PA practices at 12 months. As we have speculated previously [29, 30], food is typically a part of church culture which may facilitate implementation and maintenance of HE components. Adaptations can be made to what type of food is served and/or how food is prepared, and policies can be set regarding these practices. In contrast, for most churches, including PA requires an addition to normal practices, which might prove more difficult than simply adapting practices.

Findings from our first and third aims may appear to be in conflict. That is, analyses of continuous measures of PA and HE organizational practices revealed moderate to large increases from baseline to 24 months (albeit with significant decreases from 12 to 24 months). In contrast, a relatively small proportion of all participating churches were classified as maintainers (7%-31%), except for HE opportunities (91%) which had high implementation at baseline. Thus, most churches, while improving, did not meet the a priori criteria that we set for what constitutes “desirable” implementation. Our definition of desirable implementation, however, was quite stringent. Our findings suggest that putting the FAN components into practice may not be feasible at the frequency we envisioned. The main priorities of churches are in the spiritual realm, and it may not be realistic, for example, for someone to share messages about PA and HE at least monthly. Furthermore, the intervention was based on a structural model of behavior change [21] which points out that focusing on structural changes (organizational practices) can make small but meaningful shifts in population behavior. This idea is also consistent with RE-AIM [8]. Although we did not measure member-level behaviors, it is notable that the mean scores for organizational practices at 24 months in this study were comparable to mean scores at 15 months in an earlier study of FAN, where we also observed changes in members’ PA and HE behaviors [22, 23].

The second aim of the paper was to examine predictors of 24-month organizational practices (controlling for baseline practices). Similar factors were associated with PA and HE 24-month practices, with a few exceptions. All four intervention characteristics were associated with both PA and HE practices (adaptability, complexity, cost/time, and relative advantage). Five inner settings characteristics were associated with both PA and HE practices (compatibility, relative priority, organizational incentive, readiness for implementation, and congregant needs), but differed slightly on structural characteristics. Specifically, smaller congregation size was associated with greater HE practices, whereas predominantly African American membership was associated with greater PA practices, although the direction and magnitude of associations were similar for both. Two characteristics of implementers were associated with PA and HE practices (self-efficacy and perceived benefits). Beliefs (valuable) were associated with PA but not HE practices, whereas a higher implementer BMI was associated with HE but not PA practices. Finally, the two implementation process characteristics (opinion leader and engaging champions) were associated with both PA and HE practices. 

Like the challenges of comparing our maintenance levels with the larger literature, it is difficult to compare the factors associated with maintenance in this study to other studies due to differences in terminology, definitions, and categorization of factors. Given this caveat, our results are generally consistent with reviews of influences on the sustainability of PA interventions in school settings [40], evidence-based public health programs in community settings [38, 41], and new programs and innovations across settings [37]. Specifically, reviews of the literature find a number of factors associated with sustainability that are consistent with our results, including adaptability, fit, benefits/need, and burden/complexity of the innovation; leadership/support and structural characteristics in the inner setting; self-efficacy/skills and perceived need/benefits among implementers; and having a champion.

An earlier paper examined factors associated with 12-month implementation in this statewide initiative [30], allowing us to compare constructs associated with 12-month versus 24-month implementation. Both sets of analyses controlled for baseline practices. Overall, we found that all four CFIR domains as well as many of the same constructs within these domains were associated with both 12- and 24-month practices, suggesting that studies should plan for long-term change at the onset of the project rather than after implementation begins. Relative advantage, relative priority, organizational rewards, implementation readiness, congregant needs, implementer self-efficacy, engaging opinion leaders, and engaging champions were predictive of practices at both time periods. Furthermore, predominantly African American congregations and smaller congregations (< 500 members) had higher levels of practices at both time periods. In terms of differences, the constructs of adaptability, complexity, cost/time, and perceived benefits proved more important for 24-month than 12-month practices. Our findings suggest that over time, once the support of a formal program is diminished, these factors may become more important as churches weigh whether the program is seen as impactful enough to justify their investment of time. In contrast, the constructs of networks and communications, new ideas being readily accepted, and the FAN Coordinator’s identification with the church were related to 12-month but not 24-month practices, suggesting that a church environment that is open with good communication is helpful for starting up a new health promotion initiative.

There are several limitations to this study. First, although we enrolled more churches than other faith-based interventions in the literature, analyses for Aims 2 and 3 were limited to a sample size of 70 churches, thus limiting our power to detect associations. We found very few differences between churches (and FAN Coordinators) retained versus lost at the 24-month assessment. Second, this study was limited to United Methodist Churches in SC, and results may not generalize to other states and other denominations. Despite this limitation, there was heterogeneity in organizational practices and maintenance, as well as in ratings of CFIR items. Third, we employed a quasi-experimental design that did not include control churches. Nonetheless, our findings in this paper are consistent with our findings in other evaluations of FAN that have used more rigorous study designs [23, 26]. Devoting resources to D&I allowed us to apply RE-AIM and the CFIR in a statewide initiative and enroll a large sample of churches so that we could examine predictors of organizational practices over time. Fourth, we relied on FAN Coordinator-reported implementation. It was not feasible to conduct site visits at 92 churches located across the state. Our two previous studies demonstrated that members also reported changes in the church environment that were highly consistent with FAN Coordinator reports [22, 23, 26, 27]. Fifth, the large number of analyses for Aim 2 increases the risk for type 1 error. The predictors of 24-month organizational practices should be viewed as exploratory for guiding future studies’ selection of potentially relevant predictors. Lastly, our initial adoption of FAN might be viewed as low and may represent churches who were particularly motivated to address health; 12% of United Methodist Churches in the state enrolled in FAN. As we’ve discussed elsewhere [31], however, few faith-based studies have reported adoption rates, and when they have, they have not calculated adoption using the population base as the denominator. More often, projects have invited a subset of their sampling frame or a convenience sample to participate, and computed adoption rates based on the incomplete denominator. We used the entire population of United Methodist Churches in South Carolina as our denominator.

## Conclusions

This study makes important contributions to the faith-based health promotion literature by including a large sample of churches and testing an ecological intervention approach. The study also contributes to the larger implementation science literature by successfully applying RE-AIM and CFIR to guide the study of maintenance in an organizational setting and to predict organizational practices. The analysis of CFIR predictors was guided by a systematic process highly consistent with recommendations made by Kirk et al. [32]. We identified constructs that appeared important for PA and HE, and these findings were generally consistent with research in other settings, providing useful information to researchers and practitioners working in faith-based settings. It is encouraging that at 24 months, churches had significantly healthier environments for PA and HE than prior to training, and that these changes from pre-training to 24 months were moderate to large in magnitude. Furthermore, 24-month maintenance of FAN ecological components for churches implementing at 12 months compares favorably with prior research in other organizational settings. We believe that using a community-engaged approach to developing FAN [42], using a flexible and adaptive intervention, designing the intervention for dissemination and maintenance from its inception [43], engaging community health advisors to deliver training and technical assistance to churches, and employing an ecological model that targeted policy, systems, and environmental change all contributed to the promising implementation and maintenance findings.

## Supplementary Information

**Additional file 1. **Consolidated Framework for Implementation Research (CFIR) Domains, Constructs, and Items Assessed in the Faith, Activity, and Nutrition (FAN) Dissemination and Implementation Study, Along with Time Administered to FAN Coordinator.

## Data Availability

Because data are from church leaders in a defined state and denomination, the data could make a church and thus FAN Coordinator identifiable based on other characteristics collected. Thus, data are not included in a repository but are available from the corresponding author on reasonable request.

## Abbreviations

FAN: Faith, Activity, and Nutrition
CFIR: Consolidated Framework for Implementation Research
RE-AIM: Reach, Efficacy/Effectiveness, Adoption, Implementation, Maintenance
D&I: Dissemination and implementation
PA: Physical activity
HE: Healthy eating
SC: South Carolina

## References

[1] Campbell MK, Hudson MA, Resnicow K, Blakeney N, Paxton A, Baskin M (2007). Church-based health promotion interventions: evidence and lessons learned. Annu Rev Public Health.

[2] Levin J (2014). Faith-based partnerships for population health: Challenges, initiatives, and prospects. Public Health Rep.

[3] Olson LM, Reis J, Murphy L, Gehm JH (1988). The religious community as a partner in health care. J Community Health.

[4] Pew Research Center. In U.S., Decline of Christianity Continues at Rapid Pace. 2019. https://www.pewforum.org/2019/10/17/in-u-s-decline-of-christianity-continues-at-rapid-pace/. Accessed June 1, 2021.

[5] Pew Research Center. U.S. Religious Landscape Survey: Religious Affiliation. 2008. https://www.pewforum.org/2008/02/01/u-s-religious-landscape-survey-religious-affiliation/. Accessed June 1, 2021.

[6] Yeary KH, Klos LA, Linnan L (2012). The examination of process evaluation use in church-based health interventions: a systematic review. Health Promot Pract.

[7] Klesges LM, Estabrooks PA, Dzewaltowski DA, Bull SS, Glasgow RE (2005). Beginning with the application in mind: designing and planning health behavior change interventions to enhance dissemination. Ann Behav Med.

[8] Glasgow RE, Vogt TM, Boles SM (1999). Evaluating the public health impact of health promotion interventions: the RE-AIM framework. Am J Public Health.

[9] Harden SM, Gaglio B, Shoup JA, Kinney KA, Johnson SB, Brito F (2015). Fidelity to and comparative results across behavioral interventions evaluated through the RE-AIM framework: a systematic review. Syst Rev.

[10] Scheirer MA, Santos SL, Tagai EK, Bowie J, Slade J, Carter R (2017). Dimensions of sustainability for a health communication intervention in African American churches: a multi-methods study. Implement Sci.

[11] Blackman KC, Zoellner J, Berrey LM, Alexander R, Fanning J, Hill JL (2013). Assessing the internal and external validity of mobile health physical activity promotion interventions: a systematic literature review using the RE-AIM framework. J Med Internet Res..

[12] Compernolle S, De Cocker K, Lakerveld J, Mackenbach JD, Nijpels G, Oppert JM (2014). A RE-AIM evaluation of evidence-based multi-level interventions to improve obesity-related behaviours in adults: a systematic review (the SPOTLIGHT project). Int J Behav Nutr Phys Act.

[13] Schlechter CR, Rosenkranz RR, Guagliano JM, Dzewaltowski DA (2016). A systematic review of children's dietary interventions with parents as change agents: Application of the RE-AIM framework. Prev Med.

[14] Bhuiyan N, Singh P, Harden SM, Mama SK (2019). Rural physical activity interventions in the United States: a systematic review and RE-AIM evaluation. Int J Behav Nutr Phys Act.

[15] Galaviz KI, Harden SM, Smith E, Blackman KC, Berrey LM, Mama SK (2014). Physical activity promotion in Latin American populations: a systematic review on issues of internal and external validity. Int J Behav Nutr Phys Act.

[16] King DK, Shoup JA, Raebel MA, Anderson CB, Wagner NM, Ritzwoller DP (2020). Planning for implementation success using RE-AIM and CFIR frameworks: A qualitative study. Front Public Health.

[17] Dunn CG, Wilcox S, Saunders RP, Kaczynski AT, Blake CE, Turner-McGrievy GM (2021). Healthy Eating and Physical Activity Interventions in Faith-Based Settings: A Systematic Review Using the Reach, Effectiveness/Efficacy, Adoption, Implementation. Maintenance Framework Am J Prev Med.

[18] Bopp M, Peterson JA, Webb BL (2012). A comprehensive review of faith-based physical activity interventions. Am J Lifestyle Med.

[19] Tristao Parra M, Porfirio GJM, Arredondo EM, Atallah AN (2018). Physical activity interventions in faith-based organizations: A systematic review. Am J Health Promot.

[20] Lancaster KJ, Carter-Edwards L, Grilo S, Shen C, Schoenthaler AM (2014). Obesity interventions in African American faith-based organizations: a systematic review. Obes Rev.

[21] Cohen DA, Scribner RA, Farley TA (2000). A structural model of health behavior: a pragmatic approach to explain and influence health behaviors at the population level. Prev Med.

[22] Saunders RP, Wilcox S, Baruth M, Dowda M (2014). Process evaluation methods, implementation fidelity results and relationship to physical activity and healthy eating in the Faith, Activity, and Nutrition (FAN) study. Eval Program Plann.

[23] Wilcox S, Parrott A, Baruth M, Laken M, Condrasky M, Saunders R (2013). The Faith, Activity, and Nutrition program: a randomized controlled trial in African-American churches. Am J Prev Med.

[24] National Cancer Institute. Evidence-Based Cancer Control Programs (EBCCP),The Faith, Activity, and Nutrition (FAN) Program. https://ebccp.cancercontrol.cancer.gov/programDetails.do?programId=10977999. Accessed June 1, 2021.

[25] Damschroder LJ, Aron DC, Keith RE, Kirsh SR, Alexander JA, Lowery JC (2009). Fostering implementation of health services research findings into practice: a consolidated framework for advancing implementation science. Implement Sci.

[26] Wilcox S, Saunders RP, Kaczynski AT, Forthofer M, Sharpe PA, Goodwin C (2018). Faith, Activity, and Nutrition randomized dissemination and implementation study: Countywide adoption, reach, and effectiveness. Am J Prev Med.

[27] Saunders RP, Wilcox S, Jake-Schoffman DE, Kinnard D, Hutto B, Forthofer M (2019). The Faith, Activity, and Nutrition (FAN) Dissemination and Implementation Study, Phase 1: Implementation monitoring methods and results. Health Educ Behav.

[28] Rural Health Information Hub. Rural Health Models and Innovations, Faith, Activity, and Nutrition. 2018. https://www.ruralhealthinfo.org/project-examples/1011. Accessed June 1, 2021.

[29] Wilcox S, Saunders RP, Jake-Schoffman D, Hutto B (2020). The Faith, Activity, and Nutrition (FAN) Dissemination and Implementation Study: 24-month organizational maintenance in a countywide initiative. Front Public Health.

[30] Wilcox S, Jake-Schoffman DE, Saunders RP, Kinnard D, Kaczynski AT, Hutto B (2021). Predictors of implementation in the Faith, Activity, and Nutrition dissemination and implementation study: application of the Consolidated Framework for Implementation Research (CFIR) in a statewide initiative. Transl Behav Med.

[31] Hutto B, Saunders RP, Wilcox S, Jake-Schoffman DE, Bernhart JA, Dunn CG, et al. Pathways of influences leading to adoption of the Faith, Activity and Nutrition (FAN) program in a statewide initiative. Eval Program Plann. 2021;87:101941. 10.1016/j.evalprogplan.2021.101941 33773182

[32] Kirk MA, Kelley C, Yankey N, Birken SA, Abadie B, Damschroder L (2016). A systematic review of the use of the Consolidated Framework for Implementation Research. Implement Sci.

[33] Glasgow RE, Harden SM, Gaglio B, Rabin B, Smith ML, Porter GC, et al. RE-AIM planning and evaluation framework: Adapting to new science and practice with a 20-year review. Front Public Health. 2019;7(64). 10.3389/fpubh.2019.00064 PMC6450067 30984733

[34] Sharpe PA, Wilcox S, Stucker J, Kinnard D, Bernhart J, James KL (2020). Community health advisors' characteristics and behaviors, role performance, and volunteer satisfaction in a church-based healthy eating and physical activity intervention. J Community Health.

[35] Cohen J (1992). A power primer. Psychol Bull.

[36] Wilson KM, Brady TJ, Lesesne C, NCCDPHP Work Group on Translation. An organizing framework for translation in public health: the Knowledge to Action Framework. Prev Chronic Dis. 2011;8(2):A46. PMC3073439 21324260

[37] Wiltsey Stirman S, Kimberly J, Cook N, Calloway A, Castro F, Charns M (2012). The sustainability of new programs and innovations: a review of the empirical literature and recommendations for future research. Implement Sci.

[38] Shelton RC, Cooper BR, Stirman SW (2018). The sustainability of evidence-based interventions and practices in public health and health care. Annu Rev Public Health.

[39] Herlitz L, MacIntyre H, Osborn T, Bonell C (2020). The sustainability of public health interventions in schools: a systematic review. Implement Sci.

[40] Cassar S, Salmon J, Timperio A, Naylor PJ, van Nassau F, Contardo Ayala AM (2019). Adoption, implementation and sustainability of school-based physical activity and sedentary behaviour interventions in real-world settings: a systematic review. Int J Behav Nutr Phys Act.

[41] Scheirer MA (2005). Is sustainability possible? A review and commentary on empirical studies of program sustainability. Am J Eval.

[42] Wilcox S, Laken M, Parrott AW, Condrasky M, Saunders R, Addy CL (2010). The Faith, Activity, and Nutrition (FAN) program: design of a participatory research intervention to increase physical activity and improve dietary habits in African American churches. Contemp Clin Trials.

[43] Brownson RC, Jacobs JA, Tabak RG, Hoehner CM, Stamatakis KA (2013). Designing for dissemination among public health researchers: Findings from a national survey in the United States. Am J Public Health.

