# Supplementary material for: The Faith, Activity, and Nutrition (FAN) dissemination and implementation study: changes in and maintenance of organizational practices over 24 months in a statewide initiative
Source: Int J Behav Nutr Phys Act. 2022 Mar 2;19:23. doi: 10.1186/s12966-022-01253-9 (PMC8889739; doi:10.1186/s12966-022-01253-9)
Supplement: Supplementary file 1 — Additional file 1. Consolidated Framework for Implementation Research (CFIR) Domains, Constructs, and Items Assessed in the Faith, Activity, and Nutrition (FAN) Dissemination and Implementation Study, Along with Time Administered to FAN Coordinator. [file 12966_2022_1253_MOESM1_ESM.docx]

Additional file 1. Consolidated Framework for Implementation Research (CFIR) Domains, Constructs, and Items Assessed in the Faith, Activity, and Nutrition (FAN) Dissemination and Implementation Study, Along with Time Administered to FAN Coordinator

| **Construct** | **Description** | **Sample items & source** | **When administered** |
| --- | --- | --- | --- |
| **Domain 1: Intervention/Program Characteristics** | | | |
| Adaptability | The degree to which an intervention can be adapted, tailored, refined, or reinvented to meet local meets | The healthy eating parts of FAN can be adapted to fit my church. [[1](#_ENREF_1)]  The physical activity parts of FAN can be adapted to fit my church. [[1](#_ENREF_1)] | PT, 12M, 24M |
| Complexity | Perceived difficultly of implementation based on duration, scope, disruptiveness, centrality, steps to implement | The healthy eating parts of FAN are easy to use. [[1](#_ENREF_1)]  The physical activity parts of FAN are easy to use. [[1](#_ENREF_1)]  The healthy eating parts of FAN are clear and understandable. [[1](#_ENREF_1)]  The physical activity parts of FAN are clear and understandable. [[1](#_ENREF_1)] | PT, 12M, 24M  (12M, 24M not assessed for Pastor) |
| Cost | Costs associated with implementing the intervention, including investment, supply, and opportunity costs | The healthy eating parts of FAN are expensive to implement  The physical activity parts of FAN are expensive to implement.  The healthy eating parts of FAN take a great deal of time to implement.  The physical activity parts of FAN take a great deal of time to implement. | PT, 12M, 24M |
| Relative advantage | The perceived advantage of implementing the intervention versus an alternative program | FAN is more effective than other health programs your church has used. [[1](#_ENREF_1), [2](#_ENREF_2)] | 12M, 24M |
| **Domain 2: Inner Setting** | | | |
| Structural characteristics | The social architecture, age, maturity, and size of an organization | Does the church have an active health ministry?  Membership roll of church (#)  Predominant race of congregation  Change in pastor over study period  Tenure of current pastor | C |
| Culture | Norms, values, and basic assumptions of a given organization | You have a sense of personal responsibility for improving congregant health. [[3](#_ENREF_3)]  You are open to change in practices that impact congregants. [[4](#_ENREF_4)] | BL |
|  |  | Your pastor has a sense of personal responsibility for improving congregant health. [[3](#_ENREF_3)]  Your pastor is open to changes in church practices. [[4](#_ENREF_4)] | BL |
| Networks & communications | The nature and quality of webs of social networks and nature and quality of formal and informal communications within an organization | You have good working relationships with lay leaders in your church. [[1](#_ENREF_1)]  You actively share information and knowledge with your church. [[1](#_ENREF_1)]  You involve members when decisions are made that affect them. [[4](#_ENREF_4)]  There is very little tension and conflict between members in your church. [[4](#_ENREF_4)] | BL |
|  |  | Your pastor and church leaders actively share information and knowledge with each other. [[1](#_ENREF_1)]  Leaders in your church involve members when decisions are made. [[4](#_ENREF_4)]  Your pastor has good working relationships with other church leaders. [[1](#_ENREF_1)]  There is very little tension and conflict between members in your church. [[4](#_ENREF_4)] | BL |
| Implementation climate   - Tension for change | The capacity for change, shared receptivity to an intervention, and the extent to which use of the intervention will be rewarded, supported, and expected within the organization | Tension for change  New ideas are readily accepted in your church. [[4](#_ENREF_4)]  Leaders in your church like to keep to established, traditional ways of doing things. [[4](#_ENREF_4)] | BL |
| - Compatibility |  | Compatibility  Using FAN fits well with the way I like to work. [[1](#_ENREF_1)]  FAN matches the priorities of our church. [[5](#_ENREF_5)] | PT, 12M, 24M |
| - Relative priority |  | Relative priority  The health ministry is as important as the spiritual ministry in your church. | BL, 12M, 24M |
|  |  | Relative priority  The health ministry is as important as the spiritual ministry in your church. | 12M, 24M |
| - Organizational incentives & rewards |  | Organizational incentives & rewards  You are recognized in your church for carrying out the healthy eating parts of FAN. [[6](#_ENREF_6)]  You are recognized in your church for carrying out the physical activity parts of FAN. [[6](#_ENREF_6)] | 12M, 24M |
| Readiness for implementation   - Leadership engagement - Available resources | Tangible and immediate indicators of organizational commitment to its decision to implement and intervention | Leader engagement:  Your pastor encouraged congregants to embrace the healthy eating parts of FAN. [[5](#_ENREF_5)]  Your pastor encouraged congregants to embrace the physical activity parts of FAN. [[5](#_ENREF_5)]  Available resources:  You received enough training to carry out the healthy eating parts of FAN in your church. [[3](#_ENREF_3), [4](#_ENREF_4)]  You received enough training to carry out the physical activity parts of FAN in your church. [[3](#_ENREF_3), [4](#_ENREF_4)] | 12M, 24M |
| Congregant needs and preferences | The extent to which the intervention takes into consideration congregant preferences and reactions | The healthy eating parts of FAN have been well received by most of the congregants. [[2](#_ENREF_2)]  The physical activity parts of FAN have been well received by most of the congregants. [[2](#_ENREF_2)] | 12M, 24M |
| **Domain 3: Characteristics of Individuals involved with implementation (i.e., implementers)** | | | |
| Beliefs about the intervention | Attitudes toward and value placed on the intervention as well as familiarity with facts, truths, and principles related to the intervention | The healthy eating parts of FAN are valuable for our church.  The physical activity parts of FAN are valuable for our church. | PT, 12M, 24M |
| Self-efficacy | Individuals’ beliefs in their own capabilities to execute courses of action to achieve implementation goals | You have the skills that are needed to make the healthy eating changes for FAN work. [[5](#_ENREF_5)]  You have the skills that are needed to make the physical activity changes for FAN work. [[5](#_ENREF_5)]  I am confident that I can (12M & 24M: will be able to continue to) make the healthy eating changes for FAN. [[5](#_ENREF_5)]  I am confident that I can (12M & 24M: will be able to continue to) make the physical activity changes for FAN. [[5](#_ENREF_5)] | PT, 12M, 24M |
| Perceived benefits | The phase an individual is in during progression toward skilled, enthusiastic, and sustained use of intervention | I think my church will benefit (12M & 24M: has benefited) from the healthy eating changes for FAN. [[5](#_ENREF_5)]  I think my church will benefit (12M & 24M: has benefited) from the physical activity changes for FAN. [[5](#_ENREF_5)]  In the long run, it will be worthwhile for me if my church (12M & 24M: continues to) makes healthy eating changes. [[5](#_ENREF_5)]  In the long run, it will be worthwhile for me if my church (12M & 24M: continues to) makes physical activity changes. [[5](#_ENREF_5)] | PT, 12M, 24M |
| Individual identification with organization | How individuals perceive the organization and their relationship and commitment to it | You want to perform to the best of your ability for your church. [[4](#_ENREF_4), [6](#_ENREF_6)]  You feel a strong sense of commitment to your church. | BL |
| Other personal attributes | Other personal traits of the implementer | Has your church held any health promotion efforts?  Gender [[7](#_ENREF_7)]  Age [[7](#_ENREF_7)]  Education [[7](#_ENREF_7)]  How long have you been a member of your church?  Have you led or co-led any health promotion efforts at your church or elsewhere? | BL |
|  |  | Meeting guidelines for fruit and vegetable intake [[8](#_ENREF_8)]  Meeting guidelines for physical activity [[7](#_ENREF_7)]  Self-rated health [[7](#_ENREF_7)]  Body mass index [[7](#_ENREF_7)] | BL, 12M, 24M |
| **Domain 4: Implementation Process** | | | |
| Engaging   - Opinion leaders - Champions | Attracting and involving appropriate individuals in the implementation. Includes opinion leaders, champions, and external change agents | Opinion leaders:  Leaders in your church are actively involved in the healthy eating activities for FAN. [[1](#_ENREF_1)]  Leaders in your church are actively involved in the physical activity activities for FAN. [[1](#_ENREF_1)]  Champions:  There is at least one person in your church who is a champion for the healthy eating parts of FAN. [[2](#_ENREF_2)]  There is at least one person in your church who is a champion for the physical activity parts of FAN. [[2](#_ENREF_2)] | 12M, 24M |
| Executing | Carrying out the implementation according to plan | Fidelity & completeness of implementation as assessed by implementation measures | BL, 12M, 24M |

FAN = Faith, Activity, and Nutrition. C = reported from church conference prior to training, BL = baseline (pre-training), PT = immediate post-training, 12M = 12 months, 24M = 24 months.

Data sources (CFIR items were adapted to reference the church, church leaders, and components of FAN):

1. Cook JM, et al. Measurement of a model of implementation for health care: toward a testable theory*.* Implement Sci. 2012;7:59.

2. Thaker S, et al. Program characteristics and organizational factors affecting the implementation of a school-based indicated prevention program*.* Health Educ Res. 2008;23:238-48.

3. Helfrich CD, et al. Organizational readiness to change assessment (ORCA): development of an instrument based on the Promoting Action on Research in Health Services (PARIHS) framework*.* Implement Sci. 2009;4:38.

4. Patterson MG, et al. Validating the organizational climate measure: links to managerial practices, productivity and innovation*.* J Organ Behav. 2005;26:379-408.

5. Holt DT, et al. Readiness for organizational change: the systematic development of a scale*.* J Appl Behav Sci. 2007;43:232-255.

6. Fernandez ME, et al. Developing measures to assess constructs from the Inner Setting domain of the Consolidated Framework for Implementation Research*.* Implement Sci. 2018;13:52.

7. Centers for Disease Control and Prevention. Behavioral Risk Factor Surveillance System Survey Questionnaires. 2009 [cited 2019 August 13]; Available from: <http://www.cdc.gov/brfss/questionnaires.htm>.

8. Resnicow K, et al. Body and soul. A dietary intervention conducted through African-American churches*.* Am J Prev Med. 2004;27:97-105.
